# Mechanistic considerations and biomarkers level in nickel-induced neurodegenerative diseases: An updated systematic review

**DOI:** 10.1016/j.ibneur.2022.07.005

**Published:** 2022-07-31

**Authors:** Chidinma Promise Anyachor, Donatus Baridoo Dooka, Chinna Nneka Orish, Cecilia Nwadiuto Amadi, Beatrice Bocca, Flavia Ruggieri, Marta Senofonte, Chiara Frazzoli, Orish E. Orisakwe

**Affiliations:** a African Centre of Excellence for Public Health and Toxicological Research (ACE-PUTOR), University of Port Harcourt, PMB, 5323 Port Harcourt, Rivers State, Nigeria; b Department of Anatomy, College of Health Sciences University of Port Harcourt, PMB, 5323 Port Harcourt, Rivers State, Nigeria; c Department of Experimental Pharmacology & Toxicology, Faculty of Pharmacy, University of Port Harcourt, PMB, 5323 Port Harcourt, Rivers State, Nigeria; d Department of Environment and Health, Istituto Superiore di Sanità, Rome, Italy; e Department for Cardiovascular, Endocrine-Metabolic Diseases, and Aging, Istituto Superiore di Sanità, Rome Viale Regina Elena, 29900161 Roma, Italy

**Keywords:** Nickel, Environmental exposure, Neurotoxicity, Oxidative stress, Mitochondrial dysfunctions, Biomarkers

## Abstract

The environment has been implicated to be a strong determinant of brain health with higher risk of neurodegeneration. The drastic rise in the prevalence of neurodegenerative diseases (NDDs) including Alzheimer’s disease (AD), Parkinson’s disease (PD), autism spectrum disorder (ASD), multiple sclerosis (MS) etc., supports the idea that environmental factors may play a major role in NDDs aetiology. Nickel is one of the listed environmental metals reported to pose a serious threat to human health. This paper reported available studies on nickel level in NDDs covering both animal and human studies. Different databases were searched for articles reporting the main neurotoxicity mechanisms and the concentration of nickel in fluids and tissues of NDDs patients compared to controls. Data were extracted and synthesized by ensuring the articles were related to nickel and NDDs. Various mechanisms were reported as oxidative stress, disturbances in mitochondrial membrane potential, trace elements homeostasis destabilization, etc. Nickel was found elevated in biological fluids as blood, serum/plasma and CSF and in the brain of NDDs, as a consequence of unintentional exposure thorough nickel-contaminated air, food, water, and skin contact. In addition, after exposure to nickel, the concentration of markers of lipid peroxidation were increased, while some antioxidant defence systems decreased. Thus, the reduction in the exposure to nickel contaminant may hold a promise in reducing the incidence of NDDs.

## Highlights

• Environment affects brain health and neurodegenerative disorders NDDs.
• Nickel may pose a threat to human health.
• Body burden of Nickel is elevated in NDDs.
• Oxidative stress and inflammation mediated by nickel may lead to behavioural deficits.
• Reduction in exposure to Nickel may hold a promise in reducing the incidence of NDDs.

## Introduction

The ubiquity of heavy metals remains a significant public health concern. Whereas developed nations have evolved policies in regulating metal pollution, the same cannot be said for developing and sub-Saharan African nations (Selin, 2018, Centers for Disease Control and Prevention (CDC), 2019). Heavy metals seem to be multi-organ toxicants involving central nervous system (CNS), peripheral nervous system (PNS), hematopoietic, respiratory, endocrine, reproductive, renal and cardiovascular systems (Luo et al., 2020). Moreover, they are not biodegradable and persist in the environment with an established possibility of entry into the human body by inhalation, dermal and ingestion routes (Okoye et al., 2021, Amadi et al., 2021, Orisakwe et al., 2012). Nickel is a hard, ductile, silvery-white transition metal and abundant natural element that is 28th element in the periodic table. It may exist in several oxidative states (from −1 to +4); nevertheless, the + 2-oxidation state (Ni^2+^) is the most widespread in the environment and biological systems (Muñoz and Costa, 2012). Nickel forms alloys with iron, copper, chromium, and zinc used to make coins, stainless steel, jewellery, etc. Nickel can also combine with other elements such as chlorine, sulphur, and oxygen to form nickel compounds which dissolve appreciably in water. Nickel is a major player in modern metallurgies where it is used in a broad variety of metallurgical processes, such as alloy production, electroplating, in the production of nickel-cadmium batteries and as a catalyst in chemical and food industry (ATSDR, 2005, Genchi et al., 2020). Nickel nanoparticles (NPs) are among the nanomaterials mostly employed in many fields, such as catalysts, magnetic materials, biological medicines, conductive pastes, and additives to lubricant (Sharma et al., 2018). The brain is the most complex organ, and the hallmark of this complexity is the vast number of synapses which are also highly complex at the molecular level, with > 1000 genes encoding postsynaptic proteins in excitatory synapses (Kaizuka and Takumi, 2018). The brain is particularly vulnerable to assault during development hence the focus point is the knowledge of the possible toxicants that could impair normal brain development. Neurodegenerative diseases (NDDs) refer to a heterogeneous group of neurological disorders that affect distinct subsets of neurons in specific anatomical locations, and a number of studies have examined the relationship between exposure to environmental harmful substances and this abnormal development (Michalska and León, 2020, Brown et al., 2005). The symptoms of NDDs include deficit in behaviour, cognition, communication and sometimes motor skills. Notwithstanding there may be different types of NDDs, the most notable include Alzheimer’s disease (AD), Parkinson’s disease (PD), Huntington’s disease (HD), multiple sclerosis (MS), autism spectrum disorder (ASD), attention deficit disorder (ADD) along with less common diseases such as frontotemporal dementia (FTD) (Mullin et al., 2013, Young et al., 2018). Neurodegenerations involve diminution of neurons and their associated processes namely axons, dendrites, synapses with complementary progressive impairment in neuronal function (Jack et al., 2015), characterized by protein aggregation disorders (Hampel et al., 2017). Usually, NDDs are mediated by impaired autophagy, protein aggregation, inflammation, oxidative damage, genetic and epigenetic traits, derangement in mitochondrial processes, apoptosis, diminished growth factor and loss of synaptic plasticity (Jellinger, 2010). The major contributing factors to the noxious effects of excessive exposure to metals are their persistence, non-biodegradable and bioaccumulation (Ullah et al., 2020). The outcome and possible underlying mechanisms associated with exposure to metals and their mixtures is a topical area of research because systemic interactions of metals may cause either antagonistic or synergistic effect (Wu et al., 2016, Martin et al., 2021). The huge increase in neurodevelopmental disorders has stimulated interest in the evaluation of environmental risk factors in their aetiogenesis, and the exposure to neurotoxic metals (e.g., lead, mercury, cadmium, nickel and manganese) influenced some neurodevelopmental disorder such as autism **(**Ijomone et al., 2021; De Felice et al., 2015). High level of unsaturated fatty acids, high consumption of oxygen per unit weight and the relatively low level of antioxidant enzymes predisposes the brain to oxidative damage (Lewis et al., 2021). Metals upregulate amyloid precursor protein (APP), interleukin (IL)− 1β, IL-6, and tumor necrosis factor (TNF)-α*,* acetylcholinesterase (AChE) and monoamine oxidase-A (MAO) genes in NDDs (Lukiw et al., 2021, Cao et al., 2021), and in particular, the nervous system is profoundly vulnerable to nickel assault (Das et.al. 2008, Wu et al., 2016). Nickel was implicated in cognitive and behavioural deficits in rats with elevated levels of exposure to the metal (Lamtai et.al., 2020). Nickel can result in distinct neurological effects, with different brain targets and modes of action, and it can disrupt presynaptic neurotransmission.

In this paper, the toxicokinetic and mechanistic considerations of nickel in NDDs and levels of nickel in body fluids and tissues from diseased patients exposed to nickel were systematically reviewed. In addition, important research gaps and future areas of further research to elucidate the neurotoxicity of nickel have also been highlighted.

## Materials and methods

### Search strategy

The following databases Web of Science, EMBASE, PubMed and SCOPUS, were searched using the following key terms: “nickel” or “heavy metals” or “neurodegenerative & neurodevelopmental disorders” or “neurotoxicological implications of heavy metals” or “nickel and brain nitric oxide” or “nickel in Parkinson’s disease” or “nickel in Alzheimer’s disease” or “nickel and brain heme oxygen” or “cadherin in nickel neurotoxicity” or “nickel neurotoxicity” or “nickel in case-control studies” or “cross-sectional studies of nickel in neurodegenerative disorders” or “brain tissues, fluid and neurodegenerative disorders” or “neurotoxicity in brain neurochemistry” or “apoptosis in nickel neurotoxicity”, “oxidative stress and nickel”, or “oxidative stress and neurodegeneration”.

**Inclusion Criteria**: Studies on neurotoxicity of nickel, case-control studies of nickel exposure on brain parameters, original articles on neurodevelopmental disorders, implicating nickel and NDDs arising from nickel exposure from the target databases were included. Electronic search of the databases was added by hand search of available literature. Only relevant articles published in English language were included.

**Exclusion criteria:** Brain studies which did not implicate nickel were excluded. Duplicate articles from different databases were removed. Evaluation of the title, abstract and references of each article were screened to ensure eligibility. Authors independently selected studies, performed data extraction and evaluation.

## Results

**Fig. 1:**
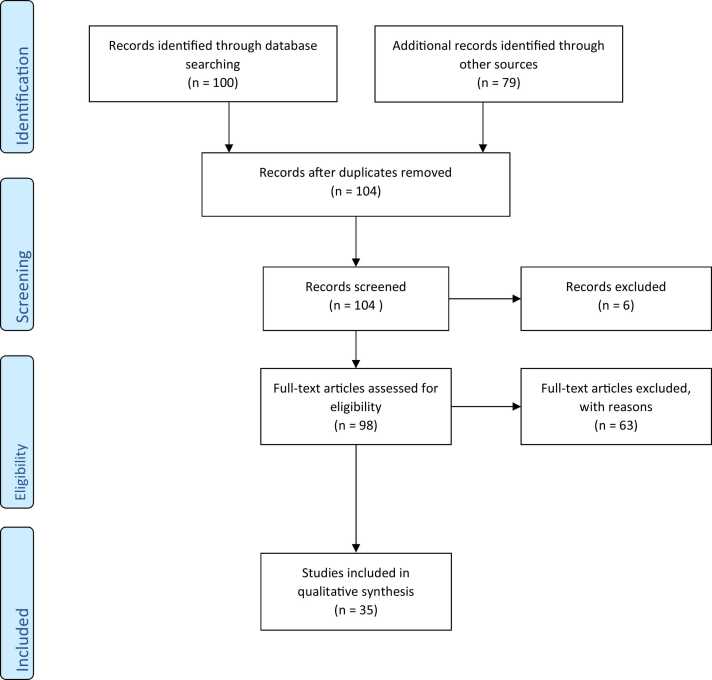
Flow chart showing the number of selected articles.

**Fig. 2:**
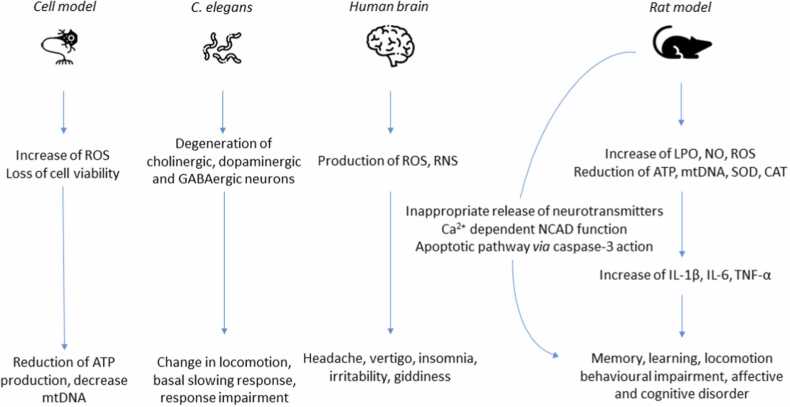
Potential mechanisms of nickel involvement in neurotoxicity ROS: reactive species oxygen; ATP: adenosine triphosphate; mtDNA: mitochondrial DNA; GABA: Gamma-aminobutyric acid; RNS: reactive nitrogen species; NCAD: N-cadherin; LPO: lipid peroxidation; NO: nitric oxide; SOD: superoxide dismutase; CAT: catalase; IL-1β: interleukin-1beta; IL-6:: interleukin-6; TNF-α: tumor necrosis factor-alpha.

The flow diagram of the complete process selection of articles is shown in Fig. 1. A total of 79 were identified through SCOPUS, Web of Science, EMBASE, while an additional 100 articles identified through PubMed. After 75 duplicates were removed, the remaining articles were 104. After title and abstract screening, a total of 6 articles were excluded. Ninety-eight (98) were further assessed for eligibility. A total number of sixty-three (63) studies were further screened out to arrive at 35 eligible articles for this work. Thirty-five (35) articles meeting the inclusion criteria were identified and used finally; they were animal and human studies that implicated nickel and neurotoxicity. Table 1 shows in vivo and in vitro animal experiments that implicated nickel in neurotoxicity and associated behavioural changes. Table 2 reported epidemiological surveys and clinical studies involving nickel exposure, comprising of country, nickel concentration in biological matrices of controls and diseased patients, number and age of diseased patients and controls, type of disorders, inferences, and mechanisms. Fig. 2 illustrates the possible mechanisms of nickel in NDDs in cell model, C. elegants, humans and rats. This figure also shows a summary of effects due to nickel exposure as memory, learning, locomotion, affective and cognitive disorders in rat models; impairment of neural cells included reduced adenosine-5′-triphosphate (ATP) production and decrease of mitochondrial DNA (mtDNA) levels in cell models; and headache, insomnia, irritability etc. in human brain. In addition, elevated nickel concentrations may be correlated to NDDs through a deregulation of physiological levels of neurotransmitters and upregulation of some other neurotransmitters. In the developing brain, nickel causes an inappropriate release of neurotransmitters that competes with calcium in their evoked release, interfering with the proper development of synaptic connections.

**Table 1:** Neurotoxicity of nickel: in vivo and in vitro animal studies.

Study No.	Experiment/Treatment	Animal model/Cell line	Result	Mechanism	Reference
1	Ability of NiO NPs (125, 250, and 500 mg/kg) to cause biochemical alterations post-acute oral exposure	Female Wistar rats	Significant inhibition of brain AChE	LPO and oxidative stress	Dumala et al. (2018)
2	Effects of short-term Ni-administration (13 mg/kg for 1 week)	Adult male rat whole brain	A statistically significant decrease in the rat brain TAS and an increase in AChE activity.	Inhibition of Na(+), K(+)-ATPase activity in rat brain	Liapi et al. (2011)
3	Evaluation of affective and cognitive behaviours, after direct and unique exposure to NiCl_2_ (300 μM)	Male Wistar rats	Memory and spatial learning disorders, affective and cognitive disorders, and oxidative stress in rats	Increased levels of NO and LPO, while CAT and SOD activities significantly decreased in the hippocampus	El Brouzi et al. (2021)
4	Neurotoxicity induced by different concentrations of NiCl2 (0.125, 0.25, 0.5, and 1 mm) for 12 hr or 0.5 mm NiCl2 for various periods (0, 3, 6, 12, and 24 hr)	Cultured cortical neurons and mouse neuroblastoma cell lines (neuro2a)	Increased ROS production and loss of cell viability both in cortisal neurons and neuro2a cell lines	Reduced ATP production and decreased mtDNA content	Xu et al. (2010b)
5	Alteration of oxidative stress and affective and cognitive behaviour after NiCl_2_ (0.25, 0.5 and 1 mg/Kg) chronic administration for 8 weeks	Male and female rats	Increased anxiety-like and depression-like behaviour, and spatial learning and memory significantly impaired only in males at 1 mg/kg of Ni	Alteration in synaptic transmission and disruption in neurotransmitters. CAT and SOD significantly decreased, while NO and LPO in the hippocampus significantly increased	Lamtai et al. (2018)
6	Comparison between the effects of NiO and NiO NPs in brain at doses of 10, 25, and 50 mg/kg intraperitoneally for 8 days on oxidative stress biomarkers	Rat brain	Increased levels of MDA, GST and CAT enzymes and decreased GSH and TAC in both NiO and NiO NPs exposed groups	Alteration of oxidative stress biomarkers	Marzban et al. (2020)
7	Ultrastructural changes to neurons in hippocampus, striatum and cortex of the brain after NiCl_2_ treatment (10 or 20 mg/kg for 4 weeks)	Rat brain	Ultrastructural alterations in neurons of hippocampus, striatum and cortex	Apoptotic pathway via caspase-3 action and perturbation of α-synuclein expression in Ni-induced neurodegeneration.	Ijomone et al. (2018b)
8	Impact of exposure to waterborne NiCl_2_ concentrations (75 and 150 μg/L).on neurobehavioral performances of rats	Rat brain	Adverse motor activities; negative geotaxis, grooming, grip strength and body rotation	Significant elevation in RONS, LPO, MPO, NO, IL-1β and TNF-α levels in cerebrum and cerebellum of treated rats	Adedara et al. (2020)
9	Effects of Ni exposure through drinking water (10 mg/L, 12 weeks) on neurobehavioral performances and dendrite complexity	Male mice	Learning and memory impairment in mice, and reduction of dendrite complexity in mouse hippocampi	Reduction of histone acetylation (especially at H3K9) and downregulation of H3K9-modulated gene expression	Zhou et al. (2021)
10	Nickel-induced neurobehavioral alterations in male and female rats after NiCl_2_ treatment (1 mg/kg for 60 days)	Male and female rats	Increased anxiety-like and depression-like behaviour in rats. Cognitive behaviour on the Morris water maze resulted compromised	LPO and NO formation with a decrease in SOD and CAT activities	Lamtai et al. (2020)
11	Neuroprotective potential of melatonin on Ni-induced neurobehavioral alterations in male and female rats	*C. elegans*	Functional changes in locomotion and basal slowing response	Increasing degeneration of cholinergic, dopaminergic and GABAergic neurons	Ijomone et al. (2020b)
12	Effects of Ni^2+^ on neurotoxic outcomes	PC12 cells	Changes in oxidative-stress-related gene expression	Downregulation of GST	Slotkin and Seidler (2010)
13	Effects of nickel concentration (10 μM) on the expression of specific neuronal differentiation markers	NT2 cells	Nickel may increase the susceptibility to neuro-psychopathology	Activation of Akt/PKB kinase pathway and increase of HIF-1a expression. Increased expression of differentiation markers MAP2 and NCAM	Ceci et al. (2015)
14	Activity of HO measured in tissues of rats after NiCl_2_ administration	Rat tissue	HO activity increased in liver, lung and brain at 17 hr after the NiCl_2_ injection	Time course, dose-effect, organ selectivity, and species susceptibility relationships for Ni induction of HO activity	Sunderman et al. (1983)
15	Impact of nickel poisoning sulphate at dose of 0.2 % on neurobehavioral functions in Wistar rats during gestation and lactation	Female rats	Impairment of spatial learning and memory performances and installation of a depressive state	Nickel poisoning during the development period causes neurotoxic effects	Kahloula et al. (2014)
16	Effect of nickel subsulfide on oxidative stress, mitochondrial membrane potential, and calcium homeostasis	Human lymphocytes	Increased generation of hydrogen peroxide (H_2_O_2_), superoxide anion (O_2_^−^) and LPO	Activation of lymphocyte death signaling pathways, excess generation of different types of oxidative stress, disturbances in mitochondrial membrane	M’Bemba-Meka et al. (2006)
18	Effect of Ni^2+^ on calcium-dependent NCAD function	Calcium titrations of NCAD in the absence and presence of fixed concentrations of Ni^2+^	Tenfold lower concentration of nickel decreases the apparent calcium-binding affinity and calcium-induced dimerization of NCAD	Nickel competes with Ca2 + for the binding sites and attenuate calcium-induced dimerization	Duke et al., 2019

NiO NPs: nickel oxide nanoparticles; AChE: acetylcholinesterase; TAS: total antioxidant status; Na(+)/K(+)-ATPase: sodium–potassium adenosine triphosphatase, NO: nitric oxide; LPO: lipid peroxidation; CAT: catalase; SOD: superoxide dismutase; mtDNA: mitochondrial DNA; MDA: malondialdehyde; GST: glutathione-s-transferase; GSH: glutathione; TAC: total antioxidant capacity; RONS: reactive oxygen and nitrogen species; MPO: myeloperoxidase; IL-1β: interleukin-1beta; TNF-α: tumor necrosis factor-alpha; C. elegans: Caenorhabditis elegans; GABA: Gamma-aminobutyric acid; Akt/PKB: protein kinase B or Akt; HIF-1α: hypoxia-inducible-factor-1α; MAP2: Microtubule-associated protein2; NCAM; neural cell adhesion molecule; HO: heme oxygenase; NCAD: N-cadherin

**Table 2:** Neurodegenerative diseases (NDDs) associated with nickel exposure: epidemiological evidences.

Study No.	Country		Nickel concentration Mean ± sd (range)		Sample	Subjects No.		Age in years Mean ± sd (range)		Disorder	Inference	Mechanism	Reference
		**Case**		**Control**		**Case**	**Control**	**Case**	**Control**				
1	**Russia**	161 µg/L		64 µg/L	Plasma	12	12	65	68	PD	Significant increased nickel levels in PD (p < 0.01)	Formation of oxygen free radicals	Johansson et al. (2007)
2	**South Indian**	0.044 ± 0.003 µg/dL		0.034 ± 0.005 µg/dL	Serum	45	42	57.6 ± 9.0	55.6 ± 3.25	PD	Significant increased Ni levels in PD (p < 0.05)	Oxidative stress, neuronal dysfunction and apoptosis	Ahmed & Santosh, 2010
3	**Norway**	0.46 ± 0.66 µg/L		0.45 ± 0.66 µg/L	Serum	33	99	4–12 yrs	nr	PD	Not significantly increased nickel levels in PD	Oxidative stress	Gellein et.al., 2008
4	**Italy**	0.53 µg/L (0.29–0.72)		0.39 µg/L (0.19–0.76)	Serum	53	124	74.5 ± 6.5 (58–86)	44.8 ± 12.7 (20–84)	AD	Higher nickel levels in AD	Significant decrease in SAC and increase in SOS in AD	Alimonti et.al., 2007
5	**Italy**	0.53 µg/L (0.41–0.77)		0.39 µg/L (0.19–0.76)	Serum	71	124	65.5 ± 9.4 (41–81)	44.8 ± 12.7 (20–84)	PD	Higher nickel levels in PD	Significant decrease in SAC and increase in SOS in AD	Alimonti et.al., 2007
6	**Italy**	0.81 µg/L (0.41–1.37)		0.39 µg/L (0.19–0.76)	Serum	60	124	38.5 ± 10.4 (24–66)	44.8 ± 12.7 (20–84)	MS	Higher nickel levels in MS	Significant decrease in SAC and increase in SOS in AD	Alimonti et.al., 2007
7	**Saudi Arabia**	0.18 ± 0.02 μg/mL		0.42 ± 0.03 μg/mL	Hair	65	77	9.0 ± 0.3	7.2 ± 0.7	ASD	Lower nickel levels in children with ASD	Decrease of excitability of CNS by nickel	Al-Ayadhi (2005)
8	**Saudi Arabia**	0.01 ± 0.03 μg/mL		0.42 ± 0.03 μg/mL	Hair	8	77	6.0 ± 0.8	7.2 ± 0.7	ADD	Lower nickel levels in children with ADD	Decrease of excitability of CNS by nickel	Al-Ayadhi (2005)
9	**Saudi Arabia**	0.06 ± 0.008 μg/mL		0.42 ± 0.03 μg/mL	Hair	2	77	10.2 ± 0.9	7.2 ± 0.7	AS	Lower nickel levels of Ni in children with AS	Decrease of excitability of CNS by nickel	Al-Ayadhi (2005)
10	**Saudi Arabia**	0.35 ± 0.2 μg/mL		0.42 ± 0.03 μg/mL	Hair	2	77	9.0 ± 2.0	7.2 ± 0.7	RS	Lower nickel levels in children with RS	Decrease of excitability of CNS by nickel	Al-Ayadhi (2005)
11	**Germany**	nr		nr	CSF	33	101	65.1 ± 12.9	44.8 ± 17.3	PD	Increased nickel levels in PD	Increased activity of ACS	Lucio et al. (2019)
12	**North Carolina**	52 ± 25 ng/g		36 ± 23 ng/g	FC	14	15	88	78	PD	Accumulation of nickel in AD brain		Szabo et al. (2016)
13	**China**	3.66 ng/mL (3.05–4.16)		3.19 ng/mL (2.61–4.00)	Serum	106	105	29.3 ± 5.6	30.65 ± 4.6)	SZ	Excess of nickel potentially associated with increased risk of developing SZ	Formation of free radicals in tissues	Cao et al. (2019)
14	**India**	0.862 µg/mL		0.454 µg/mL	Blood	40	40	nr	nr	PD	Metals, including nickel, might play a role in PD	Chronic metabolic disruption	Gupta et al. (2017)
15	**India**	2.99 ± 0.76 μg/L		1.53 ± 0.59 μg/L	Blood	225	125	56.8 ± 8.82 (35–70)	57.2 ± 7.97 (35–70)	PD	Nickel levels significantly increased in PD (p < 0.001)	Increase in oxidative stress and generation of ROS leading to neuron degeneration	Verma et al. (2016)
16	**USA**	nr		nr	Air samples	425	nr	30–55	30–55	PD	Airborne metals exposure associated with PD		Palacios et al. (2014)
17	**Germany**	2.2 ± 0.48		2.2 ± 0.83	CSF	36	42	67 ± 11.0	65.5 ± 13.1	PD	No significant changes in nickel levels in PD		Maas et al., 2018

AD: alzheimer disease; PD: parkinson disease; MS: multiple sclerosis; ASD: autism spectrum disorder; ADD: attention deficit disorder; AS: Asperger’s syndrome; RS: Rett’s syndrome; SZ: schizophrenia; CSF: cerebrospinal fluid; CNS: central nervous system; SOS: serum oxidative status; SAC: serum antioxidant capacity; ACS: acetyl-CoA synthetase; FC: frontal cortex; nr: not reported.

## Discussion

There are a number of eligible studies reporting the levels of nickel in the body or in brain of different NDDs as PD, AD, MS, ASD, Asperger’s syndrome (AS), Rett’s Syndrome (RS) and Schizophrenia (SZ) (Table 2). Several researchers have reported increased levels of nickel in serum/plasma as well as in blood samples from various ages and sexes of PD and AD cases respect to control populations (Johansson et.al., 2007, Alimonti et.al., 2007, Verma et.al., 2016; Forte et al., 2005). In CSF samples, Lucio et.al. (2019) reported increased nickel level in PD patients when compared with healthy controls, whilst Maass et al. (2018) did not observed a difference in nickel CSF levels between PD patients and controls, but ascertained nickel among others sufficiently classified PD patients. Szabo *et.al.,* (2016) analysed nickel concentration in post-mortem samples of frontal cortex (FC) and ventricular fluid (VF) from AD cases and non-demented elderly subjects. The AD cases had elevated (but not significantly) nickel levels in FC (52 ng/g) respect to the non-demented elderly controls (36 ng/g); in addition, nickel concentration in VF from control samples were 12 ng/mL and increased to 19.9 ng/mL in VF of AD cases (Szabo et.al., 2016). On the contrary, according to other surveys there was a decline in nickel levels in hair samples of autistic children respect to controls (Al-Ayadhi, 2005) or comparable levels in serum of PD cases and controls (Gellein et al., 2008). The neurotoxicity of nickel depends on the route of exposure and has a number of possible mechanisms including disruption of trace elements (as iron and calcium) homeostasis, impaired activity of enzymes, disruption in neurotransmitters, disturbances of energy metabolism, and induction of oxidative stress and mitochondrial oxidative stress, to name a few, as reported in Table 1 and as described in the following sections.

### Nickel exposure and toxicokinetic

Inhaled nickel can circumvent the blood-brain barrier and reach the olfactory neurons, constituting a potential pathway into the brain. Absorption occurs through the olfactory epithelium with a subsequent migration to primary olfactory neurons in the glomeruli of the bulbs, as well as a slow migration to secondary and tertiary olfactory neurons (Tjalve and Henriksson, 1999, Marchetti, 2014). The inhibitory effect of nickel on ATPase activity is followed by neurological disorders, convulsion, and coma. Also, accidental ingestion has been shown to cause left homonymous hemianopsia (ocular effect) (Sunderman et al., 1983, Sunderman et al., 1988). In the occupational setting, inhalation is the main route of nickel-induced toxicity. This way of exposure may also be significant in non-occupational settings among handlers of stainless steel and nickel-plated products, where allergic contact dermatitis predominates (Sinicropi et al., 2010). Occupational exposure of several million workers worldwide has given rise to high levels of nickel in blood, urine and body tissues, especially in the lungs (Vasiluk et al., 2019). Both workers occupationally exposed to nickel (Åkesson and Skerfving, 1985) and individuals who unintentionally consumed nickel from contaminated water have reported neurologic effects (Sunderman et.al., 1988). Nickel competes with copper at the albumin site forming complexes with amino acids and small polypeptides and is transported in the blood potentially leading to high concentrations in the brain (Briffa *et.al.,* 2020). Nickel is principally sequestered in the liver via calcium channels whereas renal handling and faecal excretion constitute the major routes of elimination (ATSDR, 2017, 2017, Briffa et.al., 2020). Other routes of elimination include hair, skin, saliva, sweat and milk (ATSDR, 2017, 2017). Unlike arsenic, nickel is neither methylated nor does it require any metabolism before urinary excretion (excretion half-time of 24–28 h) (Sunderman, 1989, Haber et al., 2017). It seems to be a general agreement that nickel does not bioaccumulate in humans (Haber et al., 2017).

### Oxidative, nitrosative stress and mitochondrial dysfunction in nickel neurotoxicity

Divalent nickel itself is not a good free radical generator, and nickel-induced oxidative stress is rather weak. Nickel(II) is the only oxidation state compatible with water and this makes nickel compounds redox stable and inert to reduction or oxidation in aqueous solution under physiological or even pathophysiological conditions. However, depleting glutathione and oxidatively activating various transcription factors as the nuclear factor κB (NF-κB), the AP-1 proteins, and the hypoxia inducible factor-1α (HIF-1α) cannot be ignored as possible indications of oxidative stress (Das et al., 2019). In addition, antioxidant treatment provides protection against nickel -induced neurotoxicity via decreased oxidative stress (Das et al., 2008, Xu et al., 2010a). The most well-known damaging effect of nickel is the lipid peroxidation (LPO) resulting from oxidative stress. A study by Repetto et al. (2010) found that nickel affect the process of Fe-H_2_O_2_-mediated LPO. Administration of NiCl_2_ male Wistar rats resulted in an increase in the reduced glutathione content (GSH), glutathione-S-transferase (GST), glutathione reductase (GR), LPO, H_2_O_2_ generation, with a concomitant decrease in the activity of glutathione peroxidase (GPx) (Prasad et al., 2006). Several studies reported oxidative damage in brain of rats exposed to nickel with significantly induced increase of LPO, and reduction of GSH level as well as the decrease of superoxide dismutase (SOD) and catalase (CAT) activities (Ijomone et al., 2020a, Wang et al., 2020, De Sousa et al., 2019, Deniz and Altun, 2020). In treated male Wistar albino rats, nitric oxide (NO) and LPO levels in whole brain tissue and plasma increased following nickel sulfate (NiSO_4_, 6 H_2_O) treatment (Ridnour et al., 2004, Das and Saha, 2010). Another recent study reported an elevation in the NO level and a corresponding reduction in antioxidant activities in adult male rats, and these alterations were ameliorated in the ameliorative potential of Nω-nitro-L-arginine (L-NA) - a NO synthase inhibitor - treated rats (Ijomone et al., 2021).

The NO can react with the superoxide radical (O_2_^-^) to form the peroxynitrite (ONOO^-^) and this last may cause brain mitochondrial dysfunction via a separate mechanism such as the LPO (Brookes et al., 1998, Guan et al., 2007) revealed nickel(II) chloride induces jurkat cells apoptosis via NO generation, mitochondrial depolarization and reduction of bcl-2 expression. It is important to note that the reaction of NO with ONOO^-^ forms N_2_O_3_, which may result in nitrosative stress; therefore, a fine balance between oxidative and nitrosative stress must exist in the cell in order to maintain a normal physiological state (Espey et al., 2000).

Moreover, relationship between nickel and oxidative stress can be the result of displacement and mobilization of copper from albumin and other proteins or peptides (particularly those of neuronal importance) (Briffa et al., 2020). It is also possible that Ni as a transition metal disturbs the homeostasis of the iron ion, and any perturbation of iron utilization may induce the formation of ROS, especially the inactivation of metabolic enzymes in mitochondria (He et al., 2020, Mena et al., 2015). Moreover, the binding of Ni(II) with histidyl residues of peptides and proteins might induce effects similar to those produced by Fe(II) or Cu(II) mediated Fenton-reactions (Torreilles et al., 1990). In addition, the divalent nickel may compete with the transport of divalent iron into cells via the divalent metal transporter 1 (DMT1); thus this transporter can be possibly involved in nickel uptake into the brain (EFSA, 2020). Moreover, nickel can also compete with iron sites on iron-containing enzymes like the hydroxylases, which are involved in HIF-1α modification (EFSA, 2020).

Nickel causes deficits in neurobehavioural performance in rodents and neuronal cell toxicity in vivo and in vitro, and these effects are associated with oxidative stress and disturbance of mitochondrial aerobic metabolism evidently involving HIF-1α (EFSA, 2020). Nickel also causes significant decrease in mtDNA level, which aggravates oxidative stress through declining replication of mtDNA and transcription of mtDNA encoded genes for respiratory chain (Xu et.al.*,* 2010a). Mitochondria are considered to be the major sites producing ROS and excess ROS will induce oxidative damage to unsaturated fatty acids, proteins and DNA in the mitochondria (Lin and Beal, 2006, Xu et al., 2009, Xu et.al, 2010b; He et al., 2014) observed that after nickel exposure the activity of the iron sulfur assembly proteins (ISCU1/2) responsible for mitochondrial respiration and energy production was suppressed leading to a consequent inactivation of the iron–sulfur clusters (ISCs)-containing metabolic enzymes. Moreover, deficiencies in ISC-containing metabolic enzymes not only hamper energy metabolism but also promote ROS generation by promoting abnormal mitochondrial electron transfer (Napoli et al., 2006). The oxidative stress effect was also recently investigated using Ni-NPs (Horie et al., 2011, Iqbal et al., 2021). In particular, Ni-NPs own a higher solubility than fine particles and release more Ni2 + per weight than fine particles; thus, their neurotoxicity might be greater than that of fine particles (Horie et al., 2011). Ni-NPs are stated to be more toxic than bulk forms because of their larger surface area to volume ratio and are reported to provoke toxicity through ROS generation, which leads to the upregulation of NF-κB and promotes further signaling cascades (Iqbal et al., 2021).

### Nickel and neurobehavioral disorders

Intraperitoneal injection of nickel compromises neurobehavioral performances and ultrastructural changes in neurons of the hippocampus, striatum and cortex of rats with cognitive and motor behaviors compromised and markers of oxidative stress significantly altered (Ijomone et al., 2018a). Chronic nickel exposure may alter oxidative stress markers (CAT, SOD, NO and LPO) in the hippocampus of Ni-treated rats (Lamtai et al., 2018). The hippocampus is a crucial brain region involved in the regulation of learning, memory processes, and emotion. These findings were comparable to previous reports on the association of Ni exposure with memory retention and increased the escape latency using the Morris water maze (MWM) (Kahloula et al., 2014, He et.al, 2013). Specifically, nickel increases the hippocampal level of thiobarbituric acid reactive substances (TBARS) (markers of LPO), and decrease SOD and CAT activities which may culminate in increased anxiety-like and depression-like behaviour in rats (Lamtai et al., 2020). This last study performed histopathological analyses of CA3 pyramidal neurons observing neuronal loss, cellular disorganization, and shrinkage in nickel treated rats of both genders compared to their normal controls (Lamtai et al., 2020, El Brouzi et al., 2021) demonstrated that intracerebral injection of NiCl_2_ at the right hippocampus of the male Wistar rat causes anxiety and depression disorders and lead to cognitive impairment, and again the mechanism was the increase in LPO and NO levels accompanied by a significant decrease in SOD and CAT activities in the hippocampus. In addition, Ni^2+^ exposure can inhibit and stimulate release of dopamine and also inhibit glutamate NMDA (N-methyl-D-aspartate) receptors (Martínez-Martínez et al., 2020). Therefore, molecules that constrain the oxidative stress potentially protect neuronal cells and subsequently prevent neurobehavioral disturbances, as demonstrated by melatonin administered to rats exposed to the metal (Lamtai et al., 2020, Rao et al., 2009) pointed out that the supplementation of vitamin E with nickel and chromium to mice exhibited protective effect in the ovary.

Moreover, nickel has a neuromodulator role with interference on AChE release (Liapi et al., 2011, Dumala et al., 2018). The rat brain AChE activity was found significantly increased when male Wistar rats were treated with nickel and antioxidant L-cysteine (Cys) separately, whilst it tended to change to control levels by the co-administration of nickel and Cys (Liapi et al., 2011). Similarly, inhibition of brain AchE of treated rats at the high dose of NiO nanoparticles and alteration of antioxidant enzymes were found by Dumala et al. (2018). Other mechanisms for adverse developmental actions were that nickel inhibits cell proliferation, decreases mitochondrial dehydrogenase activity, stimulates hexosaminidase release, blocks neuronal calcium channel to cause developmental neurotoxicity mediated by disruption of the gene expression of the glutamate receptors, protein kinase C (PKC) isoforms and PKC regulators in different types of nerve cells (Repetto et al., 2001, Slotkin and Seidler, 2009, Slotkin and Seidler, 2010; M’Bemba-Meka et.al., 2006). Increasing degeneration of cholinergic, dopaminergic and GABAergic neurons and significant functional changes in locomotion and basal slowing response were also reported after Ni treatment in *C. elegans* (Ijomone et al., 2020b). In addition, diminished levels of serotonin, dopamine and noradrenaline in all regions of the brain have been observed after exposure to nickel (Fatehyab et al., 1980).

Although there were no remarkable changes in whole brain pathophysiology after exposure to several nickel compounds, except for some atrophy of the olfactory epithelium in rodents (Evans et al., 1995), force feeding with NiCl_2_ for 3 months resulted in severe neurological disorders, including sluggishness, abnormal breathing, impaired body temperature regulations and ataxia (American Biogenics Corporation (ABC), 1988, Gupta et al., 2008).

In humans, neurologic effects of nickel observed among shift employees who consumed nickel-contaminated water included giddiness, weariness and headache (Sunderman et al., 1988). Transient homonymous hemianopsia (intraocular effect) for 2 h has been reported upon ingestion of a single dose of nickel (NiSO_4_; 0.05 mg Ni/Kg, body wt.) (Sunderman et al.*,* 1989, Das et al., 2019).

### Nickel in Parkinson’s disease (PD)

Parkinson’s disease is a complex, multifaceted disorder that impacts both the central and peripheral nervous systems with symptoms ranging from motor dysfunction to dementia. The motor symptoms are characterized by deficit in dopaminergic transmission in the striatum due to progressive loss of dopamine neurons in the substantia nigra pars compacta and their projections to the caudate and putamen. Parkinson’s disease now termed one of the synucleinopathies is also characterized by the deposition of the protein alpha-synuclein into Lewy bodies (Benskey et al., 2016, Miller et al., 2021). In addition to the loss of nigrostriatal dopamine neurons, accumulation of alpha-synuclein into Lewy bodies is the pathological feature of PD (Benskey et al., 2016). Also, microglial inflammation associated with α-synuclein deposition has been detected in the substantia nigra of PD patients (Croisier et al., 2005). Li and coworkers demonstrated that co-incubation of protein samples with NiO-NPs increased alpha-synuclein amyloid formation and exacerbated oxidative stress, inflammation and mitochondrial-mediated apoptosis (Li et al., 2021). Molecular modelling study again found that NiO-NPs exposure resulted in structural changes of hemoglobin and heme deformation (Gamasaee et al., 2020).

Heme oxygenase-1 (Hmox-1) is cytoprotective, and its depletion has been implicated in a spectrum of human diseases. Hmox1 is a dynamic protein that can undergo post-translational and structural modifications which modulate Hmox1 function. It is believed that, since Hmox1 can traverse from the endoplasmic reticulum to other cellular compartments including the nucleus, it may play roles other than heme break down (Dunn et al., 2014). Heme oxygenase-1 has been implicated in the pathogenesis of PD (Song et al., 2018) and heme oxygenase activity in rat brain after treatment with NiCl_2_ or Ni_3_S_2_ was found to increase (Sunderman et.al., 1983).

The paper of Johansson et.al. (2007) analysed the concentrations of nickel in plasma of patients with PD from twelve samples of patients that received L dopa and twelve healthy controls, and results showed significantly elevated Ni levels in patients respect to controls (161 µg/L vs. 64 µg/L). Another case-control study, reported serum levels higher in PD patients (0.53 µg/L) than in healthy controls (0.39 µg/L) (Alimonti et al., 2007).

Nickel can interfere with copper and zinc in the synthesis of eumelanin, pheomelanin and opiomelanin. Given the low specificity of ferro chelatase for copper, nickel may displace copper in the haem group with diminished oxygen capacity. Nickel ions enter the substantia nigra causing a decrease of the iron level. Furthermore, nickel activates dopachrome oxidoreductase to attenuate dopamine levels and the catalytic effect of nickel generates oxygen free radicals that oxidize lipids to lipofuscin and destroy tissues (Johansson et.al., 2007). Sundry reports have suggested this mechanism in the onset of different neurodegenerative diseases such as AD, Progressive Supranuclear Palsy, ALS and PD (Egana et al., 2003). Nickel is known to interact with sulphur and selenium e.g., thiol and selenol groups in GSH and GPxPx (Johansson et.al., 2007).

Nickel interferes with cellular protection by binding to OH groups and impacts synthesis process of melanin, tyrosine, and dopamine (Tallkvist, 1997). Some metal ions are vital in the synthesis of the melanin and intermediates such as eumelanin’s and pheomelanin from tyrosine via dopamine (Kronstrand, 2001). Melanin stores diverse metal ions including deleterious amounts of these metallic ions (Mars and Larsson, 1999). When melanin is oxidised, associated metal ions may be released and interact with enzymes and amino acids. Oxidation may further modify the binding capacity of melanin due to fewer binding groups or change form of the ligands (Leonard et al., 1988, Baez, 1999). The increased concentration of Ni may interfere with tyrosine hydroxylase enzyme that is believed to be copper-dependent.

### Nickel in Alzheimer’s disease (AD)

The growing number of currently diagnosed AD cases, characterised by the progressive loss of cognitive function and personality changes in aging population, represents a significant public health concern (Mayeux and Schupf, 2011, Prince et al., 2015). In addition to levels of amyloid-β and Tauproteins in CSF, which can be associated with the progression of AD (Kanai et al., 1998), ACh has been employed as a veritable biomarker of AD. Furthermore, ACh is involved in numerous functions such as cognition, memory and movement (Hasselmo and Sarter, 2010). The dysfunction in ACh regulation in the brain causes neuro-psychiatric disorders such as AD, PD, progressive dementia, myasthenia gravis (MG) and SZ (Moreira et al., 2017, Tandon, 1999). The neurotoxicity of Ni can also be mediated by the inhibition of acetylcholinesterase and Na^+^, K^+^-ATPase (Liapi et.al., 2011). In a population-based cross-sectional study in Taiwan, that investigated association between dental amalgam fillings and AD, it was suggested that oral exposure to nickel might also be a cause of AD (Sun et al., 2015). Nickel exposure significantly elevated ROS and malondialdehyde (MDA) levels, disrupted the mitochondrial membrane potential, reduced ATP concentrations and decreased mitochondrial DNA (mtDNA) copy numbers and mtRNA transcript levels (He et al., 2011). The prompt occurrence of olfactory and entorhinal pathology in AD suggests the involvement of an inhalant in the aetiology of the disease since the exposure of the olfactory neurons to the environment and their direct connections to the rhinencephalon along with the systemic access permitted by alveolar entry (Jack and Holtzman, 2013, Gandy and DeKosky, 2013, DeKosky and Gandy, 2014). Emerging evidence from clinical epidemiological and neuropathological investigations have now implicated aerosolized vehicular combustion fumes (Davis et al., 2013) and second-hand smoke (Chen, 2012). The presence of diffuse amyloid plaques and inflammation in the brains of young residents of Mexico City known for very poor air quality is a cause-and-effect indicator (Calderón-Garcidueñas et al., 2004), since it is a well-known phenomenon. In fact, the transcription of the Alzheimer amyloid precursor protein gene is controlled by acute phase reactants which give rise to rapid increases in the levels of amyloid precursor protein and its metabolite Aβ, instantly following toxic injury. Few studies have investigated brain Aβ40 and Aβ42 levels in the brains of mice exposed to an inhaled toxin model of air pollution that employed exposure to atmosphere containing aerosolized nickel nanoparticles (Ni NPs) and observed a rapid elevation (within 3 h of exposure of the mice to Ni NP) in brain (DeKosky, Gandy, 2014). The neurotoxicity of nickel (He et al., 2013) is a much less studied area than other harmful effects of nickel on human health (Lightfoot et al., 2010). The knowledge that nickel absorption via inhalation or oral exposure routes can cross the placenta and accumulate in various foetal tissues, including the brain, where they may reach concentrations much higher (above 2 µg/g) than those found in maternal blood (Casey and Robinson, 1978), led to further research. In one study, the continuous exposure of the differentiating NT2 cells to 10 mM Ni (a not cytotoxic nickel dose) upregulated neuronal differentiation markers, such as neural cell adhesion molecule (NCAM), microtubule associated protein 2 (MAP2) (Ceci et.al., 2015), and also increased hypoxia-inducible-factor-1a (HIF-1a) level inducing the activation of the Akt/PKB kinase pathway. These observations were accompanied by a clear reduction of tyrosine hydroxylase (TH), a marker of dopaminergic neurons. A not cytotoxic nickel concentration may confer hypoxia on the NT2 cells to adversely modify neuronal differentiation and hamper the expression of the dopaminergic neuronal phenotype. Early life exposure to nickel may alter normal brain development that may increase susceptibility to neuro-psychopathology later in life (Ceci et al., 2015). The miscellaneous effectors of AD include Tau and amyloid-β, along with hyper activation of kinases, oxidative stress and mutations, pose difficulties in designing therapeutic modalities. Tau is a microtubule-associating protein that causes loss of affinity for microtubules and microtubule stability and ultimately dysfunctional axonal integrity (Gorantla et al., 2020). Whereas a host of metals like iron, zinc, copper, and lead, are known to modulate Tau conformation and enhance its aggregation, Ni prevents aggregation by inducing degradation of Tau. If this school of thought gathers traction, the protective role of NiCl2 and its synthetic morpholine conjugate in AD may be anticipated in near future (Gorantla et al., 2020).

### Apoptosis in nickel neurotoxicity

Elevated hippocampal and striatal levels of caspase-3 expression the main executioner enzyme in the apoptotic pathway have been reported in nickel exposed rats (Ijomone et al., 2018b, Adedara et al., 2020). Some investigations reported that nickel treatment downregulates neuronal caspase 3 expression (Deniz and Altun, 2020). Caspase 3 is an effector or executioner caspase, a pro-apoptotic biomarker of both extrinsic and intrinsic (mitochondrial) pathways (Ghavami et al., 2009). It is plausible to consider that the involvement of Ni-induced neuronal injury may be due to exaggerated apoptotic process. Anti-apoptotic agents, which downregulate neuronal caspase 3 expression, are beneficial in NDDs and traumatic brain injury TBI (Unchiti et al., 2021). One cytotoxic study of NiO-NPs against SH-SY5Y determined by caspase-9/3 activity, and expression of apoptotic Bax and Bcl-2 genes assays, demonstrated a significant increase in the mortality of SH-SY5Y cells in an apoptotic fashion suggesting that NiO-NPs may mediate the formation of electrostatic interactions with tau proteins and induction of untoward effects on neurons (Hajimohammadjafartehrani et al., 2019, Alquezar et al., 2020) examined the neurotoxic effect of nickel in Progressive supranuclear palsy (PSP) cases using two different human cell models. Results indicated that induced pluripotent stem cell (iPSC) - derived iNeurons from a MAPT mutation carrier was more sensitive to cell death induced by nickel exposure than an isogenic control line. Moreover, using an SH-SY5Y neuroblastoma cell line, nickel induced cell death by an apoptotic mechanism (Alquezar et al., 2020). All in all, it has been postulated that nickel contributed to the pathophysiology of tauopathies such as PSP by promoting tau accumulation and neuronal cell death.

### Cadherins in nickel neurotoxicity

Cadherins are calcium-dependent cell–cell adhesion molecules that are the synaptic-tag for potentiated synapses (Okada et al., 2009, Mendez et al., 2010; Dukes et.al., 2019). N-Cadherin occurs at excitatory synapses, whereas the ‘‘Synaptic tag’’ for long term potentiation. N-Cadherin (NCAD) mediates specific cell–cell adhesive events through calcium-dependent dimerization of identical proteins on adjoining synaptic membranes (Hirano and Takeichi, 2012). NCAD is associated with diverse morphological and developmental processes such as synapse formation and maintenance, neurulation, and neurite outgrowth (Shapiro et al., 2007). Since these morphological processes occur simultaneously with changes in cadherin expression, regulated modifications of the adhesive strength and selectivity of these cell adhesion molecules are critical for proper embryonic development (Yu and Malenka, 2004). Cell adhesion molecules, including NCAD, are involved in the dynamics and regulation of synaptic structure and function that directly impact induction of long-term potentiation (Bozdagi et al., 2010). The involvement of NCAD in a variety of physiological processes requires an understanding of the microenvironmental factors that influence its adhesive behaviour. Due to the established disruption on calcium channels, it is plausible to hypothesize that divalent cations present in the extracellular matrix, such as Ni^2+^, could also disrupt the binding of calcium to N-cadherin, thereby decreasing the formation of dimer, which is required for an array of physiological and biochemical processes. The high body burden of Ni^2+^ in occupationally exposed workers (Raithel et al., 1988) may attain levels as high as 7 × the Kd for Ni^2+^ binding to N-cadherin (NCAD12). Ni^2+^ competition with calcium binding strongly decreases calcium-induced dimerization at Ni concentrations found at excitatory synapses (Dukes et.al., 2019). Calcium-induced dimerization by N-cadherin is attenuated by natural and non-physiological divalent cations in the extracellular microenvironment (Dukes et al., 2019).

## Conclusions

Along with genetic and epigenetic factors, environmental exposure to metals have been considered to play a major role in neurodevelopmental disorders. Nickel exposure through air, food, water, skin contact have also been held accountable for brain pathogenesis like Alzheimer’s and Parkinson’s diseases. In this systematic review we collected evidence of nickel involvement in neurodegenerative disorders (NDDs) through in vivo and in vitro studies. Animal and human studies were able to find a potential link between increased nickel exposure and mechanisms of neurotoxicity including induction of oxidative stress and mitochondrial oxidative stress, disruption of calcium and iron homeostasis, impaired activity of enzymes, deregulation and upregulation of neurotransmitters, disturbances of energy metabolism, etc. In many studies nickel exposure provokes damage on memory, learning, locomotion, affective and cognitive processes. In addition, early life exposure to nickel may alter normal brain development that may increase susceptibility to neuro-psychopathology later in life. Further investigations are warranted to identify treatment options both pharmaceutical and behavioural (diet, antioxidants, exercise, etc.) that may efficiently attenuate against the adverse effects of this usually overlooked heavy metal in the neuronal damaging process.

## Ethical Statement

The authors declare that the work described has not involved experimentation on humans or animals.
